# Renocardiovascular Biomarkers: from the Perspective of Managing Chronic Kidney Disease and Cardiovascular Disease

**DOI:** 10.3389/fcvm.2017.00010

**Published:** 2017-03-06

**Authors:** Shinichiro Niizuma, Yoshitaka Iwanaga, Takaharu Yahata, Shunichi Miyazaki

**Affiliations:** ^1^Department of Cardiology, Nihon University Hospital, Tokyo, Japan; ^2^Division of Cardiology, Kindai University Faculty of Medicine, Osakasayama, Japan; ^3^Department of Cardiology, Yokohama Chuo Hospital, Yokohama, Japan

**Keywords:** biomarker, cardiovascular disease, cardiorenal syndrome, chronic kidney disease, end-stage renal disease

## Abstract

Mortality among the patients with chronic kidney disease (CKD) and end-stage renal disease (ESRD) remains high because of the very high incidence of cardiovascular disease (CVD) such as coronary artery disease, cardiac hypertrophy, and heart failure. Identifying CVD in patients with CKD/ESRD remains a significant hurdle and the early diagnosis and therapy for CVD is crucial in these patients. Therefore, it is necessary for the better management to identify and utilize cardiovascular (CV) biomarkers in profiling CVD risk and enabling stratification of early mortality. This review summarizes current evidence about renocardiovascular biomarkers: CV biomarkers in patients with CKD as well as with ESRD, emphasizing on the emerging biomarkers: B-type natriuretic peptide, cardiac troponins, copeptin, the biomarker of renal injury (neutrophil gelatinase-associated lipocalin), and the mineral and bone disorder hormone/marker (fibroblast growth factor-23). Furthermore, it discusses their potential roles especially in ESRD and in future diagnostic and therapeutic strategies for CVD in the context of managing cardiorenal syndrome.

## Cardiovascular (CV) Diseases in Chronic Kidney Disease (CKD)/End-Stage Renal Disease (ESRD)

Chronic kidney disease is frequently associated with a progressive decrease in glomerular filtration rate (GFR), which leads to ESRD. The number of patients with CKD as well as ESRD is increasing markedly worldwide, with poor outcomes and high cost. Cardiovascular disease (CVD) is closely associated with CKD and ESRD and is well shown as the leading cause of morbidity and mortality in the patients with CKD, most notably those on ESRD or dialysis (1). The prevalence of concomitant coronary artery disease (CAD), left ventricular hypertrophy (LVH), congestive heart failure (CHF), cardiac arrhythmia (most commonly atrial fibrillation), and valvular/vascular calcification are increased in CKD patients. Recently, the term, cardiorenal syndrome (CRS) has been introduced in an attempt to emphasize the interaction between the CV and renal systems in acute or chronic disease setting (2). CRS is a pathophysiological condition in which combined cardiac and renal dysfunction amplifies the progression of failure of the individual organs and has an extremely bad prognosis. It is more than simultaneous cardiac and renal disease. It is now necessary for us to expand our knowledge regarding its pathogenesis, prevention, and potential treatment.

## Renocardiovascular Biomarkers

A number of biomarkers have been evaluated in CVD or CKD/ESRD, and biomarkers pertinent to the CV and renal interface are defined as renocardiovascular biomarkers (3). From the perspective of pathophysiology, they are classified into (I) neurohormones (4–9), (II) metabolic hormones/peptides (10–13), (III) cardiac injury markers (14, 15), (IV) oxidative stress markers (16–21), (V) matrix-related markers (22–27), (VI) inflammatory markers (28–31), (VII) renal markers (32), and (VIII) mineral and bone disorder hormones/markers (33–41), as summarized in Table 1.

**Table 1:** **Potential renocardiovascular biomarkers**.

Biomarkers		Reference
**(I) Neurohormones**		
Natriuretic peptides (ANP, B-type natriuretic peptide (BNP), CNP, and related peptides)	BNP and its amino-terminal fragment (NT-proBNP) have become established as the most ideal markers of HF so far available	(4)

Endothelin and C-terminal-pro-endothelin-1 (CT-proET-1)	CT-proET-1 increases in CKD and may be potentially useful biomarkers of renal injury	(5)

Arginine vasopressin (AVP) and copeptin	Copeptin which is released with AVP, emerges to be a more reliable marker for HF and is also released into the circulation early after MI onset and may aid in rapid diagnosis	(6, 7)

Adrenomedullin (ADM) and mid-regional proadrenomedullin (MR-proADM)	MR-proADM, which is relatively more stable than ADM, is used to explore the prognostic power for HF related deaths, suggesting better predictability than the natriuretic peptides	(8, 9)

**(II) Metabolic hormones/peptides**		
Triiodothyronine, adrenocorticotropic hormone and cortisol	Hormonal derangements at the level of the hypothalamic-pituitary axis are often seen with the CKD or CVD	(10)

Adiponectin	Although hyperadiponectinemia is a common phenomenon in CKD and is considered to have similar beneficial effects on metabolic risk in this patient group, many recent studies have unexpectedly shown that high, rather than low, concentrations predict mortality	(11)

Leptin	Hyperleptinemia, frequently observed in CKD patients, may play a key role in the pathogenesis of complications associated with CKD such as cachexia, protein energy wasting, chronic inflammation, insulin resistance, cardiovascular (CV) damages, and bone complications. Leptin may be also involved in the progression of renal disease through its pro-fibrotic and pro-hypertensive actions	(12, 13)

**(III) Cardiac injury markers**		
Cardiac troponins (cTns)	There are accumulating evidences of the usefulness of cTns in conditions other than ACS, including HF	(14)

Heart-type fatty acid-binding protein (H-FABP)	H-FABP as well as cTns is influenced by renal function and the utilities may be somewhat limited in CKD patients	(15)

**(IV) Oxidative stress markers**		
Malondialdehyde, 8-hydroxy-2′ -deoxyguanosine, oxidized low-density lipoproteins	A number of biological markers of oxidative stress have been evaluated for CVD as well as CKD, since oxidative stress is a common mediator in pathophysiology of risk factors for the both diseases	(16)

Uric acid	Although at present, there are no definite data whether uric acid is causal, compensatory, coincidental, or it is only an epiphenomenon in CKD patients, hyperuricemia may contribute to the development and progression of CKD and also be linked to CVD	(17, 18)

Advanced glycation endproduct (AGE)	Chronic overstimulation of the AGE-receptor for AGE pathway is likely one of major contributors involved in the pathophysiology of CVD in patients with CKD	(19)

Asymmetric dimethylarginine (ADMA)	ADMA level is a marker and mediator of oxidative stress as well as endothelial dysfunction/atherosclerosis. It is accumulated in plasma during CKD and a strong predictor for the progression of CKD and CVD in CKD patients. Also in patients with HD, plasma ADMA is a strong and independent predictor of overall mortality and CV outcome	(20, 21)

**(V) Matrix-related markers**		
Matrix metalloproteinases and tissue inhibitors of MMPs	They have been increasingly linked to both normal physiology and abnormal pathology in the kidney as well as heart. However, their roles in the pathophysiology of CRS are extremely complex	(22)

Galectin-3	Galectin-3 has been associated with increased risk for morbidity and mortality in patients with HF. Also, it may be causally involved in mechanisms of tubulointerstitial fibrosis and CKD progression	(23–25)

ST2	ST2 is reflective of fibrosis and cardiac remodeling and strongly related to HF outcomes. Also, cross-sectionally, higher ST2 concentrations appear to be associated with worse kidney function in patients with CVD	(26, 27)

**(VI) Inflammatory markers**		
High-sensitive C-reactive protein (hs-CRP), cytokines and related receptors [Interleukin (IL)-1, -2, -6, -8, -18, TNF-α]	Among CKD patients, inflammatory biomarkers including hs-CRP and IL-6 correlate with known CVD and provide prognostic information, which suggests inflammation and oxidative stress may contribute to CV risk in CKD patients	(28)

Pentraxin-3 (PTX3)	Elevated systemic PTX3 levels appear to be a powerful marker of inflammatory status and a superior outcome predictor in patients with CKD. It may also provide more information on development and progression of atherosclerosis than other less specific markers such as CRP	(29)

Growth differentiation factor-15 (GDF-15)	Measurement of circulating GDF-15 provides incremental improvement in mortality risk prediction in addition to traditional risk factors as well as cTns and BNP in healthy individuals as well in patients with a spectrum of CVD including AMI and HF. Higher circulating GDF-15 is associated with incident renal outcomes and improves risk prediction of incident CKD	(30, 31)

**(VII) Renal markers**		
Neutrophil gelatinase-associated lipocalin (NGAL), kidney injury molecule-1, liver-type fatty acid-binding protein, cystatin C	In AKI and CKD, these renal biomarkers have become useful for assessment and prognostication both in plasma and in urine. They are expected to be useful in early diagnosis of renal involvement in CVD such as HF, and contrast nephropathy, and to suggest the timing of treatment initiation and its likely effectiveness	(32)

**(VIII) Mineral and bone disorder hormones/markers**		
Vitamin D/parathyroid hormone (PTH)	Serum markers such as calcium, phosphate, vitamin D and PTH are already in use in clinical practice, and increased calcium, increased phosphate, decreased (active) vitamin D, or increased PTH are reported to be associated with CVD in CKD/ESRD patients by epidemiologic data. However, the relationship between these markers and CVD in CKD patients is complex and may lack causality	(33–37)

Osteoprotegerin (OPG)	Elevated OPG levels have been associated with aortic stiffness and markers of CV dysfunction such as raised serum troponin T levels	(38)

Fetuin-A	Decreased fetuin-A levels are observed in CKD patients, especially in ESRD and are associated with the development of vascular calcification	(39)

Fibroblast growth factor-23 (FGF-23)	FGF-23 levels among CKD patients are higher than in the general population and are further elevated in the dialysis population. It may cause not only rapid progression of renal functional decline, but also the development of LVH and HF	(40, 41)

*ACS, acute coronary syndromes; AKI, acute kidney injury; CKD, chronic kidney disease; CRS, cardio-renal syndrome; CVD, cardiovascular disease; ESRD, end-stage renal disease; HD, hemodialysis; HF, heart failure; LVH, left ventricular hypertrophy; MI, myocardial infarction*.

## Emerging Renocardiovascular Biomarkers

**Figure 1:**
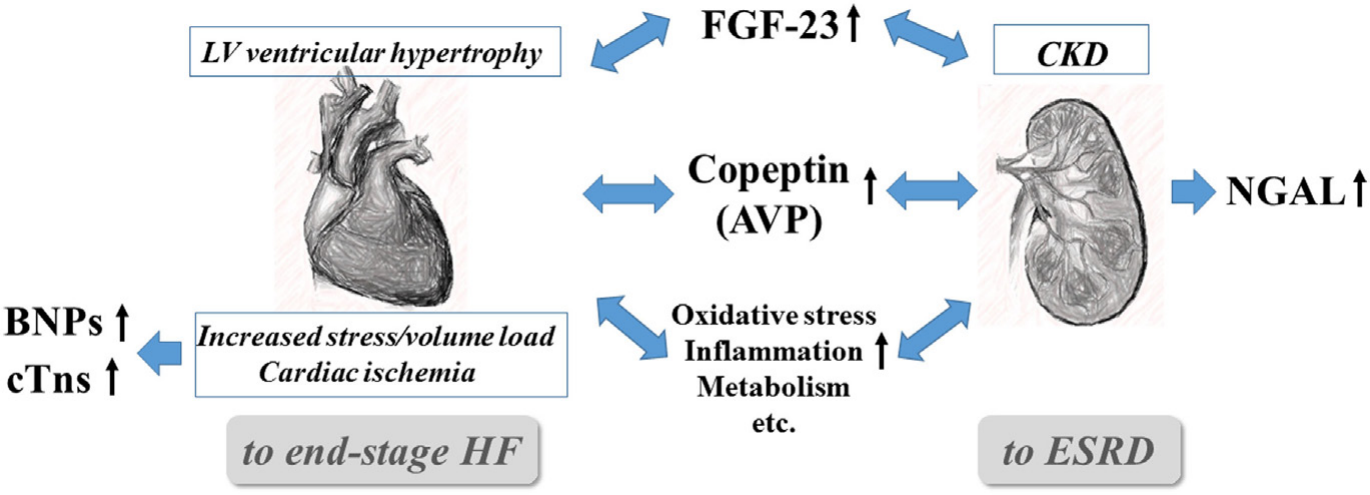
**Interrelationships and pivotal roles of the five emerging biomarkers in cardiorenal syndrome**. AVP, arginine vasopressin; BNPs, B-type natriuretic peptides (BNP and NT-proBNP), CKD, chronic kidney disease; cTns, cardiac troponins (troponin I and T); CVD, cardiovascular disease; ESRD, end-stage renal disease; FGF-23, fibroblast growth factor-23; and NGAL, neutrophil gelatinase-associated lipocalin.

Among the biomarkers listed in Table 1, the critical biomarkers in the context of managing CRS are discussed in detail, first briefly describing their pathological associations and then the evidence for their usefulness in relation to important clinical endpoints (Figure 1).

### A: B-Type Natriuretic Peptide (BNP) and Its Amino-Terminal Fragment (NT-proBNP)

Upon ventricular myocyte stretch, preproBNP is enzymatically cleaved to proBNP and released in the form of the active hormone BNP (amino acids 79–108) or an inactive fragment, NT-proBNP (amino acids 1–76, released in a1:1 ratio). The hemodynamic load (i.e., myocardial stretch) is the most important stimulus for BNP and NT-proBNP secretion, based on the results of both basic and clinical studies. Iwanaga et al. have demonstrated an excellent correlation between BNP and LV end-diastolic wall stress (EDWS) (*r*^2^ = 0.89, *P* < 0.001) in HF patients with normal creatinine levels, and they found that this relationship was more robust than any other parameter previously reported (42). Currently, both BNP and NT-proBNP are widely used as markers for a variety of CVD. However, there are differences between the two assays; NT-proBNP has a longer half-life and thus, its levels may be more stable (less sensitive to acute stress). In addition, NT-proBNP might be more dependent on renal clearance than BNP. To date, most studies have demonstrated that both are equally useful for diagnosis, management and prognosis, even in CKD and hemodialysis (HD) patients.

#### BNP/NT-proBNP vs. Renal Function

The relationship between BNP/NT-proBNP levels and renal dysfunction is complex. Any change in value may be influenced by renal dysfunction due to change in clearance. When renal dysfunction causes cardiac damage/stress, directly or indirectly, this will cause increased BNP/NT-proBNP values. It remains unclear whether, in patients with CKD and ESRD, increased plasma BNP levels might be due to more hemodynamic stimuli or might result from other factors such as anemia, obesity, and cachexia or impaired renal clearance of natriuretic peptide, despite similar hemodynamic stimuli. Multiple studies have shown an inverse moderate, but significant, correlation between estimated glomerular filtration ratio (eGFR) and BNP or NT-proBNP concentrations. Niizuma et al. have recently shown that renal dysfunction (reduced eGFR) may contribute to increased BNP levels (inverse correlation) independent of EDWS (a critical hemodynamic determinant of BNP), anemia, HF type, and BMI in a wide spectrum of HF patients with both CKD and ESRD (43). Some reports suggest that NT-proBNP is more strongly influenced with the severity of renal dysfunction than BNP, whereas others show that they are equally dependent on the renal function for their clearance. van Kimmenade et al. showed BNP and NT-proBNP had nearly identical correlations to eGFR (*r* = −0.35 and *r* = −0.3, respectively; *P* < 0.001 for both) in 165 hypertensive subjects (44). They went a step further by measuring renal fractional extraction (FE) [(renal artery concentration − renal vein concentration)/renal artery concentration] of these NPs and found that, across a range of eGFR as low as 9 mL/min/1.73 m^2^, FE for BNP and NT-proBNP diminished only modestly and correlated minimally with eGFR (*r* = 0.20–0.26). Furthermore, they found in a multivariate regression analysis that cardio-related factors such as blood pressure, LV mass, and eGFR, but not FE, were significantly associated with NPs concentrations.

#### BNP and NT-proBNP Elevations in ESRD

Among patients with HD or peritoneal dialysis (PD), considerable heterogeneity in BNP and NT-proBNP levels has been recognized and seems to increase exponentially, which may have caused some confusion in interpreting results. In ESRD or dialysis patients, the question of whether increased plasma NP levels are due to cardiac hemodynamic stimuli, accompanying comorbidities or impaired renal clearance of NPs is also a matter of controversy. Recent reports in ESRD patients suggest that the NT-proBNP/BNP ratio increases even further in patients receiving HD (45). In dialysis patients, the parameters related to the dialysis treatment itself may influence BNP and NT-proBNP concentrations, like the type of dialysis membrane and a patient’s volume status or change (46, 47). Certainly, the very high prevalence of LV structural and functional abnormalities in ESRD patients may account for the remarkable elevations. Zoccali et al. demonstrated that ANP/BNP levels were only slightly elevated in dialysis patients who had normal cardiac function and no LVH on echocardiography (48).

#### Diagnostic and Prognostic Utility of BNP and NT-proBNP in ESRD

The best cut-off values for detecting HF may need to be raised when the eGFR is less than 60 mL/min. It should be noted that the diagnostic accuracy of plasma BNP and NT-proBNP for HF is reduced in this setting, and natriuretic peptide testing for HF should be discouraged in patients especially on dialysis. Recently, Mishra et al. reported in the large CKD cohort without HF that NT-proBNP had strong associations with prevalent LVH and LV systolic dysfunction (49). Also, some studies demonstrated a close association between BNP or NT-proBNP levels and LV mass and systolic function in the ESRD population. Experimental studies suggested myocardial ischemia apart from an increased mechanical stress, upregulated BNP gene expression, and release from ventricular myocardium (50). Recently, Niizuma et al. used CAG for a thorough evaluation of disease severity and assessed the relationship between BNP and CAD in 125 patients with long-term HD (51). Plasma BNP levels showed a predictive value for CAD and were closely correlated with disease severity, and similar findings were observed also in the studies of patients with CKD. Thus, the measurement of plasma BNP levels in combination with other non-invasive investigations might help in assessing CAD involvement and aggressive management in this high-risk population. In addition to their roles in detecting LV abnormality or ischemic status, a number of studies suggest BNP testing as promising cardiac biomarkers for mortality prediction and CV risk stratification also in dialysis patients (48). The largest study by Apple et al. examined the predialysis NT-proBNP levels in 399 HD patients and showed that NT-proBNP was significantly predictive of mortality, and the area under the receiver operating characteristic curve in relation to mortality was higher with NT-proBNP than with cTnT or High-sensitive C-reactive protein (52). Also in the largest study in the PD population, patients in the highest quartile of NT-proBNP had significantly greater risk of mortality, CV death, and events after a median follow-up of 36 months (53). All of these data suggest the prognostic importance of BNP or NT-proBNP level at a single time point, irrespective of whether the measurement was taken before dialysis, after dialysis, or midweek between dialysis. In addition to CV outcomes, several studies suggested that increased BNP and NT-proBNP concentrations were associated with an increased risk for accelerated progression of CKD to ESRD (54). The mechanism may be complex and cardiac abnormalities or other comorbidities in addition to the decreased clearance may attribute to the results. In any case, it strongly suggests a dynamic interplay between the heart and the kidney as CRS.

### B: Troponins

After myocardial cell damage, cardiac troponin (cTn) T and I are released from the myocytes and their levels are detectable 3–12 h after the injury and mean time to peak cTn level is approximately 12–48 h. The concentration returns to the normal range after 5–14 days, which is four times longer than for the creatine kinase myocardial band isoenzyme (CK-MB) fraction. The increasing sensitivity of cTn assays has had a dramatic impact on the use of cTn testing, which is now an essential component of the diagnostic workup and management of acute coronary syndromes (ACS). More importantly, the test has prognostic value because it identifies ACS patients who are at substantially increased risk of death or recurrent MI (15). Also, the elevated cTn in asymptomatic individuals in the community is associated with a tripling of risk of all-cause and CV mortality as shown by a recent systematic review (55).

#### cTns in CKD/ESRD

Elevated cTns may be detected in conditions other than ACS, including HF, cardiomyopathies, myocarditis, tachyarrhythmia, and pulmonary embolism, and even after strenuous exercise in healthy individuals (14). Levels of cTns are frequently elevated in the absence of ACS among patients with renal dysfunction, specifically in 30–75% of ESRD patients. In general, the use of more sensitive cTn assays likely shows that the presence of cTn elevations in ESRD patients might be even more frequent than previously thought. Jacobs et al. found that by using a more sensitive cTnT assay, 94% of patients had cTnT elevations above the 99th percentile (56). At one time, it was considered that an elevation of the cTns in a setting of decreased creatinine clearance was not of substantial diagnostic or prognostic importance. However, the GUSTO IV trial, which included 7,033 patients with suspected ACS, indicated that an elevated cTnT level was strongly predictive of poor short-term prognosis, regardless of creatinine clearance (57). Although the important limitations were the very small number of patients with severe CKD or ESRD and its confinement to symptomatic patients, cTnT elevation had even greater prognostic importance among patients with a mild to moderate degree of CKD. Stacy et al. have showed that cTns can identify those with a poor prognosis in patients with CKD and suspected ACS by a systemic review (58). In addition, they found that cTns levels can be helpful but that their diagnostic utility is limited by varying estimates of sensitivity and specificity. In the clinical setting, serial measurements should be performed in patients CKD and suspected ACS because an exact cut-off point cannot yet be specified. Moreover, over the past decade, data have emerged to suggest that elevated levels of cTns may predict death among ESRD patients without symptoms of ACS. A recent research by Michos and colleagues have reviewed 98 studies systematically and showed the association of elevated cTns with worse prognosis in CKD patients (receiving or not receiving dialysis) without suspected ACS (59). Although the precise mechanism of death is unknown, several studies suggest that the prevalence of increased levels of cTns may correlate with increased risk of CAD. deFillippi et al. found that a high level (>median) vs. a low level of cTnT remained an independent predictor of multivessel disease (prevalence ratio, 3.7 fold) after adjustment for age and history of clinical CAD in stable patients undergoing long-term HD (60). They also suggested the potential complementary role of serum CRP, which is elevated in more than 70% of HD patients, for predicting all-cause mortality. Routine measurement of cTns may be prognostically valuable and may help frame therapeutic decisions. However, the pathophysiologic mechanisms causing random increases in cTn concentrations in patients with renal dysfunction or dialysis are still unclear. Elevation of cTn levels in ESRD patients is unlikely to be the result of decreased clearance by the failing kidney, and cTn elevation might reflect cardiac damage in non-ischemic cardiomyopathy or microvascular disease in the setting of LV hypertrophy. There is also an evidence that dialysis can affect cTn levels (cTnT is increased after HD, whereas cTnI is decreased). It should be noted again that cTn testing does not provide any insight into the mechanism of cardiac injury and that a broad differential diagnosis including renal failure, pulmonary embolism and HF is routinely considered when cTn concentration is elevated in the absence of ACS. In the recent guideline (61), measurement of cTns is recommended for the evaluation of ACS in patients with ESRD (level of evidence A). A dynamic change in cTns of ≥20% after presentation should be used to define ACS (level of evidence B). Baseline cTns can aid in defining mortality and CV risk for patients with ESRD and provide baseline levels for the subsequent comparison (level of evidence B).

#### cTns in HF

In the context of HF, evidence for a role of cTns continues to accumulate, particularly for use in risk stratification. Evidence of myocyte cell death in human myocardium has been suggested by histologic information of biopsies and more recently by the measurement of the serum cTns, which may be able to identify subclinical injury to the myocardium. Not only in ischemic HF but also in non-ischemic HF, cTns could identify a subgroup of patients with severe HF. In patients with acute decompensated HF, 6.2% were positive for cTn testing and they showed higher in-hospital mortality than those with negative test (62). During routine clinical follow-up of ambulatory patients, elevations in cTns, particularly frequently or persistently, could be associated with an increased risk of events. In addition, Sundström et al. reported that the elevated serum level of cTnI was an independent contributor to the development of HF in a community-based sample of 1,089 70-year-old men (63). Clinical significance of increased cTns concentrations in CKD patients with HF as well as those with ACS or stable CAD is unclear. Chen et al. reported the association of an elevated cTnI with HF in 293 non-ACS patients with CKD, where Stage 5 CKD did not modify it (64). Bansal et al. reported that cTnT level was strongly associated with incident HF in a prospective cohort of 3,483 people with mild to severe CKD (65). They also showed not only cTnT but also NT-proBNP was independently associated with HF. In patients with dialysis, elevated cTnT may be a more important risk factor than LV systolic dysfunction alone and adds value when used in combination with LV mass index and ejection fraction in predicting circulatory congestion. Recently, Tsutamoto et al. reported that decreased clearance *via* the kidney might contribute to the elevated cTnT in HF patients with CKD by measuring the difference of TnT concentration between coronary sinus and aortic root (66). However, it is a controversial issue and further large studies will be needed to clarify whether renal dysfunction itself or additional myocyte damage might impact the clinical outcome in HF patients with CKD in the context of CRS. Interestingly, recent studies suggest that cTnT and NT-proBNP levels independently predicted ESRD risk in the general population or patients with diabetic nephropathy and anemia (67). These results may support a link between cardiac injury and the development of ESRD. To date, the data support the concept that structural markers of myocardial damage or strain are perhaps the best prognostic markers in high-risk patients with CVD.

### C: Copeptin

The effects of arginine vasopressin (AVP) are mediated by three receptor types: V1aR, V1bR, and V2R. V1aR-dependent vasoconstriction that increases afterload, ventricular stress, and cardiac hypertrophy may result in systemic vascular resistance and a decrease in cardiac output and contractility while V2R is the receptor that governs water retention by activating the aquaporin-2 channel on the apical plasma membrane of the collecting duct cells (6). Despite its suggested importance in the pathogenesis of CVD, the evaluation of AVP secretion has been difficult because of the considerable technical issues related to AVP measurement and AVP’s short plasma half-life or instability (68). Copeptin, a glycosylated 39-amino acid long peptide, is the C-terminal part of pro-AVP and is cosecreted in equimolar amounts with together with AVP during precursor processing (7). In contrast to AVP, copeptin has a longer plasma half-life, is very stable at room temperature and easy and robust to measure. Studies in healthy subjects have shown plasma copeptin and AVP concentrations to correlate strongly over a wide range of osmolality. For these reasons, copeptin is now considered as a robust surrogate for AVP, overcoming the technical problems related to AVP dosage.

During the last few years, plasma copeptin concentration has been measured in a number of clinical investigations. Significant associations have been found between baseline copeptin level and the incidence or progression of various pathological situations such as HF, diabetes, and CKD, suggesting that AVP may play causal roles. Other studies have shown that plasma copeptin may be a useful diagnostic marker in patients with AMI, sepsis, or unexplained hyponatremia. Furthermore, the prognostic values of copeptin have already been shown in patients with HF, CAD, and with acute stroke. It should be noted that most findings as the diagnostic and prognostic marker of CVD were from subjects without renal dysfunction.

#### Copeptin in CKD/ESRD

In CKD, a few data exist evaluating copeptin concentrations, which found copeptin to be associated with renal function (69). It might be because AVP causes renal function decline, subjects with low renal function are less sensitive to the actions of AVP or increased copeptin may result from reduced renal elimination. Increased AVP concentrations in CKD have already been reported and are associated with biologic activity. A number of contributive factors such as LV dysfunction (or HF), endothelial stress, V2 receptor resistance, and diabetes might be related with its elevation. However, a recent study in a normal population showed that the close relationship between copeptin and AVP was distorted in CKD, suggesting that the peptide clearances might differ when the renal function is impaired (70). It is not conclusive and further studies are necessary. Meijer et al. examined the role of copeptin baseline values and the change in renal function in a cohort of 548 renal transplant recipients (71). Elevated copeptin concentration was associated with an accelerated renal function decline in patients with renal transplant, as seen by a decrease in GFR during a median follow-up of 3.6 years. A recent cross-sectional study describes the association of plasma copeptin levels with eGFR, urinary albumin excretion, kidney length, and renal simple cysts in a large, multicentric population-based cohort (72). There are a few studies investigating associations between plasma copeptin and CVD in CKD/ESRD. Fenske et al. have reported an increased incidence of CV events in a subgroup of ESRD patients who had elevated levels of copeptin (73), and Engelbertz et al. showed elevated copeptin as a prognostic factor, independent of serum creatinine (SCr) in patients with both CAD and CKD (74). Whether increased AVP (copeptin) secretion is prognostic or causative for CVD especially in CKD patients and copeptin may serve as a biomarker to identify CVD high-risk patients with CKD needs to be evaluated in future studies (75).

### D: Neutrophil Gelatinase-Associated Lipocalin (NGAL)

Although acute kidney injury (AKI) and CKD are conditions that substantially increase morbidity and mortality, the both diagnosis is still made with surrogate markers of GFR, such as SCr, urine output, and creatinine based estimating equations. The creatinine-base equations are an unreliable indicator during acute and chronic changes in kidney function, and new biomarkers should have the potential to identify earlier patients with AKI and CKD and in the future potentially intervene to modify outcomes. Among them, NGAL is considered one of excellent biomarkers in urine and plasma for the early prediction of AKI as well as CKD detection (32, 76).

#### NGAL in AKI/CKD

Neutrophil gelatinase-associated lipocalin (NGAL), also known as lipocalin-2, is a 25-kDa secretary glycoprotein and a member of lipocalin superfamily of proteins and has been shown to be induced rapidly in renal tubules in response to acute injury. NGAL seems to be one of the earliest kidney markers of ischemic or nephrotoxic injury in animal models and is detected in the blood and urine of humans soon after AKI. A meta-analysis by Haase et al. demonstrated that NGAL was an early predictor of subclinical AKI, with early elevations in plasma NGAL levels compared to SCr (77). Moreover, in-patient mortality was highest in those patients with elevated NGAL levels, with or without elevated SCr. In a prospective observational cohort study of 158 patients with Stage 3 or 4 CKD, it is suggested that urinary NGAL in addition to conventional established CV and renal risk factors may improve the prediction of progression to ESRD requiring renal replacement therapy (78). The role of NGAL has also been studied in the setting of post-cardiac surgery, cardiac catheterization, hemolytic uremic syndrome, and kidney transplantation.

#### NGAL in CVD

Aghel et al. recently reported that elevated admission serum NGAL levels was associated with heightened risk of subsequent development of worsening renal function (WRF) in 91 patients who admitted with acute HF (79). NGAL could be useful as an earlier marker of impending WRF during the treatment of acute HF. In patients with chronic HF, Damman et al. suggested that renal impairment is not only characterized by decreased eGFR and increased urinary albumin excretion but also by increased urinary NGAL concentration (80). Poniatowski et al. reported that predictors of serum NGAL in 150 patients with chronic HF were NYHA class, cystatin C, and eGFR in multiple regression analysis (81). NGAL might be investigated as a potential early and sensitive marker of kidney dysfunction/injury in order to select the appropriate strategy for reducing the risk in the setting of CRS.

However, recent evidence demonstrates a diversity of expression and function of NGAL. NGAL in plasma is also generated by a systemic inflammation, which induces NGAL synthesis by extrarenal tissues and the release of NGAL from neutrophils. It has been implicated not only as a marker of renal function but also as that of neutrophil activation, a protective factor against apoptosis and oxidative stress. Recent reports have suggested that NGAL could be a biomarker of atherosclerosis or CVD as well as in AKI and CKD progression (82). Increased NGAL expression in atherosclerotic plaques has been demonstrated and serum NGAL levels were significantly higher in the presence of CAD and were correlated with the severity of the disease. Also, in HF, increased myocardial expression of NGAL might be one of mechanisms for its prognostic value, independent on the coexisting renal injury. A recent study by Daniels et al. showed that plasma NGAL was a significant predictor of mortality and CVD in community-dwelling older adults, independent of traditional risk factors and kidney function, and adds incremental value to NT-proBNP and CRP (83).

Recently, among patients with CKD, urine levels of NGAL, were reported to be associated independently with future ischemic atherosclerotic events, but not with HF events or deaths (84). Higher urine NGAL may reflect a heavier burden of vascular diseases including subclinical inflammation and endothelial damage in the kidney as well as other organs. Clinical studies on the role of NGAL in ESRD patients are few. However, recent one study has showed that serum levels of NGAL in HD patients with CVD were significantly higher than those without CVD, and serum NGAL levels were independent risk factors in multivariate logistic regression analysis (85). Also in HD patients, increased serum NGAL might reflect the CV inflammation and damages. Further basic and clinical studies are warranted to elucidate the true pathogenic role of increased NGAL and to confirm the use as a biomarker for CRS.

### E: Fibroblast Growth Factor (FGF)-23

Fibroblast growth factor-23 (FGF-23) is a recently discovered member of the FGF family, involved in the regulation of the body’s calcium-phosphate metabolism (40). It increases renal phosphate excretion by reducing the expression of Na/Pi IIa cotransporter and decreases circulating calcitriol [1,25(OH)2D3] levels, leading to increased parathyroid hormone levels resulting in secondary hyperparathyroidism. FGF-23 levels gradually increase with declining renal function, starting at the very earliest stages of CKD, increasing by many orders of magnitude in ESRD. Some studies suggested that most circulating FGF-23 may be functionally intact, indicating a mechanism involving increased FGF-23 secretion as the cause of elevated FGF-23 levels. Oversecretion of FGF-23 may allow the body to maintain phosphate levels within a “physiological” range (~2.5–4.5 mg/dl) until very advanced CKD stages.

#### FGF-23 and Mortality

Elevated FGF-23 levels are associated with an increased risk of adverse outcomes, including all-cause mortality, CV events, and progression of CKD (86). Isakova et al. evaluated FGF-23 as a risk factor for adverse outcomes in a prospective 5-year study of 3,879 participants with CKD stages 2–4. They have shown that elevated FGF-23 was an independent risk factor for ESRD in patients with relatively preserved kidney function and for mortality across the spectrum of CKD (41). In a nested case–control sample of 200 subjects who died and 200 who survived during the first year taken from a prospective cohort of 10,044 incident HD patients, Gutiérrez et al. also showed that increased FGF-23 levels appear to be independently associated with mortality (87). Interestingly, some studies have described an association of FGF-23 with CV events and mortality in patients without CKD in whom the typical constellation of abnormal bone-mineral metabolism seen in CKD and ESRD is not present (88). Thus, FGF-23 may be a novel marker of CVD in patients with CKD and ESRD as well as in normal renal function, although these associations appear to be stronger in patients with CKD/ESRD. Also, they are independent of serum phosphate levels, and FGF-23 levels are a significant independent predictor among various parameters of calcium-phosphate metabolism.

#### FGF-23 and CVD

In cross-sectional studies, increased FGF-23 levels in patients with CKD were found to be associated not only with therapy-resistant secondary hyperparathyroidism but were also independently related to LVH and endothelial dysfunction after adjustment for traditional markers of calcium–phosphate metabolism. In a large study of 3,939 CKD patients, Faul et al. showed that FGF-23 was independently associated with LVH, which is an important mechanism of CVD in patients with CKD (89). In basic studies, this may be *via* the FGF receptor–dependent activation of the calcineurin-NFAT-signaling pathway independent of klotho (the co-receptor of FGF-23 only expressed in kidney and parathyroid glands). Treatment with an FGF receptor blocker is also reported to attenuate LVH. In contrast, observational studies reported conflicting results on the association of FGF-23 with arterial calcification, which is another prominent pattern of CV injury in CKD. In the largest study to date, FGF-23 was not independently associated with coronary artery calcification in patients with CKD stages 2–4 (90). These data suggest that direct effects of FGF-23 on cardiac remodeling, rather than the arterial vasculature, may underlie its association with mortality. Recently, Scialla et al. reported that higher FGF-23 level was independently associated with greater risk of CV events, particularly CHF, in a prospective cohort of 3,860 participants with CKD stages 2–4 (91). They demonstrated that elevated FGF-23 was associated more strongly with CHF than with atherosclerotic events (*P* = 0.02), and uniformly was associated with greater risk of CHF events across subgroups stratified by eGFR, proteinuria, prior heart disease, diabetes, BP control, anemia, LV mass index, and ejection fraction. Their findings suggest that FGF-23 could represent a novel mechanism of CHF that mediates at least a portion of excess CVD risk attributable to CKD. Recently, additional analysis in their study showed the association of FGF-23 with atrial fibrillation in CKD, which is frequently observed and has clinical impact in patients with CKD (92). Thus, in light of the high CV morbidity in CKD patients, as well as the failure of traditional therapeutic concepts (e.g., cholesterol-lowering or angiotensin-converting enzyme inhibitor therapy) and of non-traditional approaches (e.g., normalization of hemoglobin levels, increasing dialysis dose, and use of high-flux dialyzer), the option of pharmacological lowering of high circulating FGF-23 levels is expected to be a promising therapeutic pathway for reducing CVD in CKD and ESRD.

## Conclusion and Future Perspectives

Of great importance is recognizing the presence of CRS and appreciating the impact it can play on treatment options and survival. However, our understanding of the pathophysiology of CRS or CVD in CKD/ESRD remains relatively poor. Reliable renocardiovascular biomarkers are nowadays necessary for evaluating accurately the complex interactions that are the basis of CRS and for early diagnosis and staging of CVD and CKD/ESRD, which lead to more accurate and efficient patient management and the development of strategies for CV risk stratification and prevention in this condition. On the contrary, it is necessary for clinicians and investigators to consider the pathologic mechanisms linking heart and kidney for the interpretation and utilization in such biomarkers and to look beyond just biomarkers measured at presentation and statistical tests of association. From such an integrating viewpoint, further studies into the biomarkers will facilitate our knowledge of CRS and improve the clinical managements.

Recently, the aberrant profiles of circulating microRNAs (miRNAs) have been evaluated as emerging biomarkers for CV or renal disease. For example, miR-21 is associated with organ fibrosis and the measurement may be useful in the detection of renal or myocardial fibrosis (93, 94). Several studies have started to clarify the prognostic roles of various miRNAs in large cohorts (95). However, available data are limited especially in the context of CRS, and more studies and validation in large cohorts are necessary for establishing the clinical utility of circulating and urinary miRNAs. In addition, a multi-biomarker approach for targeting several organs or pathophysiologies is quite reasonable in this situation since CRS is a very complex disorder not of a single organ but of multi-organs including heart, vasculatures and kidney (52, 65). More integrated assessments of a multi-biomarker approach will be performed and shed light on the clinical management in CKD and ESRD patients. Another advance may be in “biomarker-guided monitoring of therapy”. An individual patient meta-analysis suggested that BNP-guided monitoring of HF therapy is associated with a significant reduction in all-cause mortality in 11 studies randomizing 2,000 patients (96). Currently, the impact of renal insufficiency on the treatment effect by BNP guiding remains uncertain. However, such trials using BNP as well as other renocardiovascular biomarkers in CKD patients will be performed not only on HF, but also on CAD or LVH.

## Author Contributions

The contribution of each author on this work was as follows: SN and YI, the primary investigator; TY, the secondary investigator (data analysis); and SM, the consultant and supervisor.

## Conflict of Interest Statement

The authors declare that the research was conducted in the absence of any commercial or financial relationships that could be construed as a potential conflict of interest.
